# World Health Organization Discontinues Its Drinking-Water Guideline for Manganese

**DOI:** 10.1289/ehp.1104693

**Published:** 2012-02-14

**Authors:** Seth H. Frisbie, Erika J. Mitchell, Hannah Dustin, Donald M. Maynard, Bibudhendra Sarkar

**Affiliations:** 1 Department of Chemistry and Biochemistry, Norwich University, Northfield, Vermont, USA; 2 Better Life Laboratories, Inc., East Calais, Vermont, USA; 3 Department of Molecular Structure and Function, Research Institute of the Hospital for Sick Children and Department of Biochemistry, University of Toronto, Toronto, Ontario, Canada

**Keywords:** drinking-water, guideline, manganese, public health, World Health Organization

## Abstract

Background: The World Health Organization (WHO) released the fourth edition of *Guidelines for Drinking-Water Quality* in July 2011. In this edition, the 400-µg/L drinking-water guideline for manganese (Mn) was discontinued with the assertion that because “this health-based value is well above concentrations of manganese normally found in drinking water, it is not considered necessary to derive a formal guideline value.”

Objective: In this commentary, we review the WHO guideline for Mn in drinking water—from its introduction in 1958 through its discontinuation in 2011.

Methods: For the primary references, we used the WHO publications that documented the Mn guidelines. We used peer-reviewed journal articles, government reports, published conference proceedings, and theses to identify countries with drinking water or potential drinking-water supplies exceeding 400 µg/L Mn and peer-reviewed journal articles to summarize the health effects of Mn.

Discussion: Drinking water or potential drinking-water supplies with Mn concentrations > 400 µg/L are found in a substantial number of countries worldwide. The drinking water of many tens of millions of people has Mn concentrations > 400 µg/L. Recent research on the health effects of Mn suggests that the earlier WHO guideline of 400 µg/L may have been too high to adequately protect public health.

Conclusions: The toxic effects and geographic distribution of Mn in drinking-water supplies justify a reevaluation by the WHO of its decision to discontinue its drinking-water guideline for Mn.

For the past 53 years, the World Health Organization (WHO) has listed manganese (Mn) as a threat to potable water. However, in the recently released fourth edition of the WHO *Guidelines for Drinking-Water Quality* (WHO 2011a), the guideline for Mn was discontinued:

The 1958 WHO *International Standards for Drinking-water* suggested that concentrations of manganese greater than 0.5 mg/l [500 µg/L] would markedly impair the potability of the water. The 1963 and 1971 International Standards retained this value as a maximum allowable or permissible concentration. In the first edition of the *Guidelines for Drinking-water Quality*, published in 1984, a guideline value of 0.1 mg/l [100 µg/L] was established for manganese, based on its staining properties.

A health-based drinking-water guideline of 500 µg/L for Mn was issued in the second edition of *Guidelines for Drinking-Water Quality*, which was published in 1993. This 500-µg/L guideline was estimated—it was not calculated:

Although no single study is suitable for use in calculating a guideline value, the weight of evidence from actual daily intake [in humans] and from studies in laboratory animals given drinking-water in which neurotoxic and other effects were observed supports the view that a provisional health-based guideline value of 0.5 mg/litre [500 µg/L] should be adequate to protect public health. (WHO 1996)

The WHO issued a more protective health-based drinking-water guideline of 400 µg/L for Mn in the third edition of *Guidelines for Drinking-Water Quality*, published in 2004. This 400-µg/L guideline was calculated from “the upper range value of manganese intake . . . identified using dietary surveys, at which there are no observed adverse effects” (WHO 2004).

However, the 400-µg/L guideline for Mn was discontinued in the fourth edition of *Guidelines for Drinking-Water Quality*, published in 2011, because the WHO (2011b) asserted that

this health-based value [400 µg/L] is well above concentrations of manganese normally found in drinking-water, [so] it is not considered necessary to derive a formal guideline value.

A review of WHO publications, peer-reviewed journal articles, government reports, published conference proceedings, and theses strongly suggests that Mn is found > 400 µg/L in drinking water or in potential drinking-water supplies in a substantial number of countries (Table 1). Affected areas include large population centers as well as small pockets of contamination that affect just a few households. In Bangladesh alone, it is likely that > 60 million people are drinking water with Mn > 400 µg/L (British Geological Survey 2001; Frisbie et al. 2002; Hasan and Ali 2010) (Figure 1).

**Table 1:** Examples of countries with documented instances of drinking water or potential drinking water sources with Mn concentrations > 400 μg/L.

Country	Type of contamination			Reference
Australia		N		Zaw and Chiswell 1999
Bangladesh		N		Frisbie et al. 2002
		A		Bhuiyan et al. 2010
Benin		U		Zogo et al. 2011
Bolivia		A		González Alonso et al. 2010
Botswana		A		Staudt 2003
Bulgaria		U		Litvinov 1962
Cambodia		U		Buschmann et al. 2007
Canada		U		Barbeau et al. 2011
Chile		U		Araya-Valenzuela and Espejo-Guasp 2003
China		N		Weng et al. 2007
Croatia		N		Štembal et al. 2005
Czech Republic		N		Kožíšek et al. 2008
East Timor		U		Michael 2006
Egypt		A		Taha et al. 2004
Ghana		U		Amoako et al. 2011
Greece		U		Kondakis et al. 1989
Honduras		U		Meeroff et al. 2007
Hungary		U		Deák et al. 1993
India		U		Ramakrishnaiah et al. 2009
Indonesia		U		Stauder and Eggers 2010
Ireland		U		Toner et al. 2003
Italy		U		Roccaro et al. 2007
Japan		A		Kawamura et al. 1941
Kenya		A		Kithiia and Ongwenyi 1997
Laos		U		Chanpiwat 2011
Lesotho		U		Pullanikkatil 2008
Lithuania		U		Gražulevicˇiene˙ and Balcˇius 2009
Madagascar		U		Rasolofonirina et al. 2004
Malaysia		A		Hasan et al. 2011
Mexico		A		Huizar-Alvarez 1997
Mongolia		N		Smedley et al. 2003
Morocco		N		Azzaoui et al. 2002
Myanmar		U		Aye et al. 2010
Nepal		U		Mahat and Shrestha 2008
New Zealand		U		Daughney 2003
Nigeria		A		Gbadebo and Taiwo 2011
Pakistan		A		Majidano and Khuhawar 2009
Poland		U		Bray and Olan´czuk-Neyman 2003
Romania		A		Dima et al. 2006
Russia		U		Serikov et al. 2009
Rwanda		A		Julius 2011
Saudi Arabia		U		Alabdula’aly et al. 2011
Slovakia		U		Barloková and Ilavský 2009
Sri Lanka		U		Institute for Global Environmental Strategies 2007
Sweden		U		Ljung et al. 2007
Taiwan		U		Shyu et al. 2011
Thailand		U		Promma et al. 2002
Turkey		A		Demirel 2007
Uganda		U		Taylor and Howard 1994
United Kingdom		U		Homoncik et al. 2010
United States		U		Groschen et al. 2008
Vietnam		U		Buschmann et al. 2007
Zambia		A		Kasonde 1993
Zimbabwe		A		Meck et al. 2009
Abbreviations: A, Mn from anthropogenic sources; N, Mn from natural sources; U, Mn from unspecified sources.				

**Figure 1:**
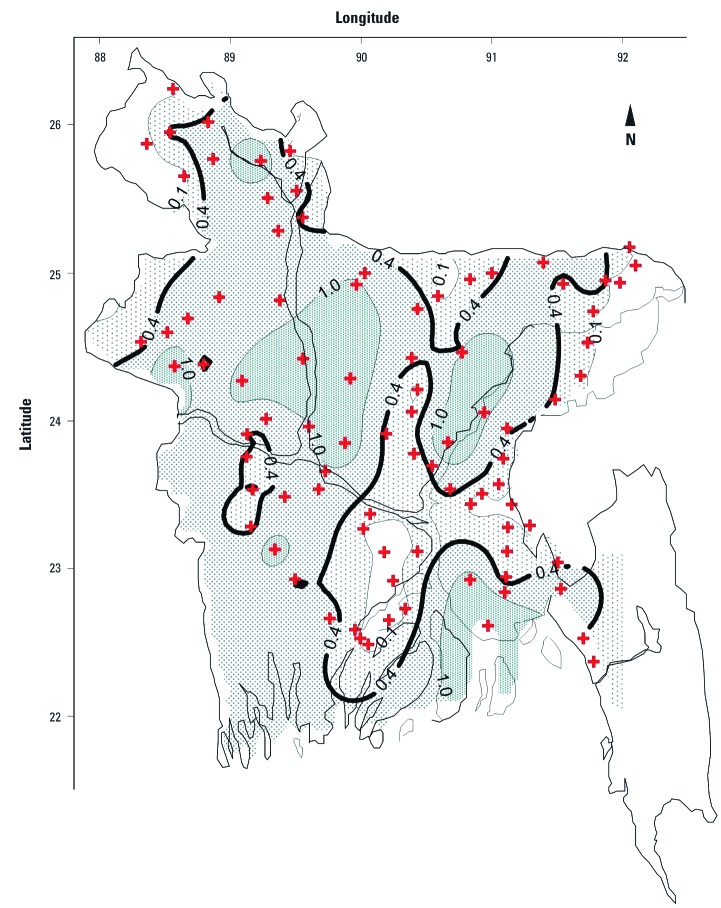
Contour map of Mn concentration (mg/L) in Bangladesh’s drinking well water. Each sampling location is labeled with a plus symbol. The thick black contour line represents the former WHO health-based drinking-water guideline of 0.4 mg/L (400 μg/L). Adapted from Frisbie et al. (2002).

In spite of the recent claim that Mn in drinking water is not found above 400 µg/L and is not a threat to human health, the WHO (1996) previously stated that Mn in drinking water from Greece and Japan greatly exceeded 400 µg/L and caused significant neurological damage in humans:

An epidemiological study was conducted in Greece” where “the levels of manganese were 3.6–14.6 µg/litre in the control area and 81–282 µg/litre and 1,800–2,300 µg/litre in the test areas [2,300 µg/L is 5.75 times greater than the 400 µg/L guideline]. The authors concluded that progressive increases in the manganese concentration in drinking-water are associated with progressively higher prevalences of neurological signs of chronic manganese poisoning.

In an epidemiological study in Japan, adverse effects were seen in humans consuming manganese dissolved in drinking-water, probably at a concentration close to 28 mg/litre [28,000 µg/L is 70 times greater than the 400 µg/L guideline]. The manganese was derived from 400 dry-cell batteries buried near a drinking-water well. A total of 16 cases of poisoning were reported, the symptoms including lethargy, increased muscle tone, tremor, and mental disturbances.

This tragedy in Japan underscores the fact that the drinking-water guidelines must apply to both natural and anthropogenic sources of contamination. Drinking water guidelines are used to decide whether or not water from a particular source is safe to drink. Most drinking-water guidelines issued by the WHO are for industrial pollutants such as dichlorodiphenyltrichloroethane (DDT), tetrachloroethylene (PCE), or vinyl chloride (WHO 2011b), but guidelines are also issued for toxins such as arsenic that may be of either natural or anthropogenic origin. Industrial pollution, such as the improper disposal of dry-cell batteries or other toxic wastes, can easily yield Mn concentrations well above those “normally found in drinking-water” (WHO 2011b) and cause significant harm to public health.

Mn is a powerful neurotoxin that causes learning disabilities and deficits in intellectual function in children (Barlow 1983; Bouchard et al. 2007, 2011; Collipp et al. 1983; Ericson et al. 2007; Henn et al. 2011; Kim et al. 2009; Menezes-Filho et al. 2011; Riojas-Rodríguez et al. 2010; Takser et al. 2003; Wasserman et al. 2006; Woolf et al. 2002; Wright et al. 2006; Yousef et al. 2011) and manganism and Mn-induced parkinsonism in adults (Aschner et al. 2009; Barceloux 1999; Beuter et al. 1999; Calne et al. 1994; Erikson et al. 2005; Guilarte 2010; Lucchini et al. 2009; Perl and Olanow 2007; Rodríguez-Agudelo et al. 2006; Sikk et al. 2007; Standridge et al. 2008) and children (Sahni et al. 2007), as well as compulsive behaviors, emotional lability, hallucinations, and attention disorders (Bowler et al. 1999; Kawamura et al. 1941; Kondakis et al. 1989; Solís-Vivanco et al. 2009). In addition, high maternal Mn levels are associated with low fetal birth weight (Gražulevičiene et al. 2009; Zota et al. 2009) and increased infant mortality (Hafeman et al. 2007; Spangler and Spangler 2009). Mn in drinking water also has been correlated with all-cause cancer rates (Spangler and Reid 2010).

Many key studies documenting the neuro-toxic effects of Mn in children (Bouchard et al. 2007, 2011; Henn et al. 2011; Wasserman et al. 2006) and adults (Huang 2007; Lucchini et al. 2009; Perl and Olanow 2007) were published within the past 5 years. This research was not yet available in 2004 when the WHO set its health-based guideline of 400 µg/L. Based on these new toxicity findings, several authors have argued that the 400 µg/L health-based guideline was too high to adequately protect human health and recommended a reexamination of the Mn guideline (Ljung and Vahter 2007).

Examples of drinking water or potential drinking-water supplies with Mn concentrations > 400 µg/L can be found worldwide. Knowledge about the toxic effects of Mn, particularly with human exposure through drinking water, has grown considerably over the past 10 years. The WHO drinking-water guidelines are used by many governments to help set regu-la-tions to protect the public health of their citizens. In the absence of a WHO guideline on Mn, governments and other stakeholders must take into consideration the likelihood of exposure to Mn through drinking water for their populations as well as research results on toxic effects of Mn in setting their own -regulations for Mn.
